# Decode the Stable Cell Communications Based on Neuropeptide-Receptors Network in 36746 Tumor Cells

**DOI:** 10.3390/biomedicines10010014

**Published:** 2021-12-22

**Authors:** Yining Liu, Min Zhao

**Affiliations:** 1 The School of Public Health, Institute for Chemical Carcinogenesis, Guangzhou Medical University, Guangzhou 511436, China; yiningliu.pku@gmail.com; 2 School of Science, Technology and Engineering, University of the Sunshine Coast, Maroochydore Dc, QLD 4558, Australia

**Keywords:** single-cell RNAseq, neuropeptide receptor, cancer genomics, data integration

## Abstract

Background: As chemical signals of hormones, neuropeptides are essential to regulate cell growth by interacting with their receptors to achieve cell communications in cancer tissues. Previously, neuropeptide transcriptome analysis was limited to tissue-based bulk expression levels. The molecular mechanisms of neuropeptides and their receptors at the single-cell level remain unclear. We conducted a systematic single-cell transcriptome data integration analysis to clarify the similarities and variations of neuropeptide-mediated cell communication between various malignancies. Methods: Based on the single-cell expression information in 72 cancer datasets across 24 cancer types, we characterized actively expressed neuropeptides and receptors as having log values of the quantitative transcripts per million ≥ 1. Then, we created the putative cell-to-cell communication network for each dataset by using the known interaction of those actively expressed neuropeptides and receptors. To focus on the stable cell communication events, we identified neuropeptide and downstream receptors whose interactions were detected in more than half of all conceivable cell-cell interactions (square of the total cell population) in a dataset. Results: Focusing on those actively expressed neuropeptides and receptors, we built over 76 million cell-to-cell communications across 70 cancer datasets. Then the stable cell communication analyses were applied to each dataset, and about 14 million stable cell-to-cell communications could be detected based on 16 neuropeptides and 23 receptors. Further functional analysis indicates these 39 genes could regulate blood pressure and are significantly associated with patients’ survival among over ten thousand The Cancer Genome Atlas (TCGA)pan-cancer samples. By zooming in lung cancer-specific clinical features, we discovered the 39 genes appeared to be enriched in the patients with smoking. In skin cancer, they may differ in the patients with the distinct histological subtype and molecular drivers. Conclusions: At the single-cell level, stable cell communications across cancer types demonstrated some common and distinct neuropeptide-receptor patterns, which could be helpful in determining the status of neuropeptide-based cell communication and developing a peptide-based therapy strategy.

## 1. Introduction

Malignant tumors seriously threaten human life and health and currently still maintain a high morbidity and mortality rate worldwide. According to worldwide statistics in 2020, there will be approximately 19.3 million new cancer cases and approximately 10.0 million deaths [1]. Although radiotherapy and chemotherapy combined with surgery can reduce cancer mortality, existing anti-tumor drugs can kill tumor cells while having more significant side effects on normal cells. Therefore, developing new anti-tumor drugs with low toxicity and side effects is important to reduce the recurrent rate and improve the survival rate. Peptide drugs have attracted attention in developing anti-tumor drugs due to their safety and specificity [2].

A neuropeptide is an endogenous peptide that exists in the central nervous system and participates in the function of the nervous system [3]. As short peptide signals, neuropeptides can regulate the functional activities of various system organs and cells in the body. With high activity and diversity, neuropeptides can be involved in regulating a large number of biological and physiological processes, such as smooth muscle contraction, blood pressure, and inflammation [4]. At the same time, neuropeptides are also considered to be influential cell growth factors for many cell types, including cancer cells [5]. The role of neuropeptides involves all essential processes in cancer initiation and metastasis. Therefore, understanding the mechanism of neuropeptide in cancer development may provide clues to identify effective neuropeptide-based anti-tumor drugs. For example, a novel peptide combination consisting of four synthetic neuropeptide analogs of vasoactive intestinal peptide (VIP), bombesin, substance P and somatostatin have potent anticancer activity in vitro and in vivo [6].

To date, tissue-based gene expression technology could measure the averaged gene expression in a sample containing millions of cells. Although this can identify differentially expressed genes and transcripts in different cell populations, many subtle differences between cells in the same tissue sample will be overlooked. Combined with high-throughput cell isolation, Single-cell RNA-sequencing (scRNASeq) is a new technology for high-throughput sequencing of mRNA at the single-cell level [7]. scRNASeq can effectively explore cancer heterogeneity that cannot be determined by tissue-based transcriptome analysis [7]. Although there are accumulated associations of neuropeptides and cancer, we do not know much about the composition and function of neuropeptides at the single-cell level. To fill this knowledge gap, we explored the stable expression of neuropeptides and their receptors in various cancers at the single-cell level. We might provide novel mechanisms for the development of neuropeptide-based anti-tumor drugs.

## 2. Materials and Methods

### 2.1. The Cancer-Related scRNAseq Datasets

To compare the neuropeptide in different cancers, we utilized the pre-computed database CancerSEA [8]. By focusing on single-cell transcriptome data, CancerSEA collected 72 datasets from various cancers. In CancerSEA, the data processing started from strict quality control and normalization procedures, and finally Transcripts Per Kilobase Million (TPM) was used to quantify all expression values. Our analysis is based on the log2 transformed TPM expression value extracted from 72 CancerSEA datasets. In addition, CancerSEA also calculated fourteen cell states based on those marker genes of the key cell states related to cancers, including angiogenesis, apoptosis, cell cycle, differentiation, DNA damage, DNA repair, epithelial-mesenchymal transition (EMT), hypoxia, inflammation, invasion, metastasis, proliferation, and stemness. In the CancerSEA database, they have the expression data for both protein-coding and long non-coding transcripts. To focus on reliable protein-coding genes, we downloaded the single-cell expression profile about protein-coding transcripts and the corresponding 14 cell functional states for all 72 datasets.

### 2.2. The Pan-Cancer Expression Profile for Neuropeptide and Receptor at the Single-Cell Level

Our previous work about the copy number changes of neuropeptides collected a total of 93 human neuropeptide precursors in cancers [9]. Specifically, there are three primary sources of data for these neuropeptides: a keyword-based search for the NCBI RefSeq protein database; NeuroPep, a comprehensive neuropeptide database [10]; and two published manuscripts on neuropeptide evolution [11,12]. Using known human ligand-receptor information [13], we mapped all the known human neuropeptides to the receptors. In total, 260 human neuropeptide-receptor pairs with 226 unique genes were listed in Table S1. In our previous work [9], we also defined four fully connected functional modules with ten or more genes, which were also be used in this study (Table S2). The fully connected functional modules are helpful to cluster the cell with similar neuropeptide receptor signals.

By mapping to all the downloaded expression data from CancerSEA, all 226 neuropeptides and receptor genes’ expression data were extracted. We defined genes that are actively expressed for each data set if the log2 transformed TPM value of a neuropeptide or its receptor is equal to or greater than 1. In this way, we can summarize the active expression status of all neuropeptides and receptors in each cell. Then, the relationships between cells were further established through the known interaction between neuropeptides and their receptors (Table S1). Finally, seventy neuropeptide-based cell communication maps were extracted from 70 datasets (Table S3).

### 2.3. The Stable Cell Communication through Active Expressed Neuropeptides and Receptors at the Single-Cell Level

To define stable cell communication, we calculated a stable cell communication ratio based on the active expressed neuropeptides and receptors in a dataset:

(1) $$s=x/y^{2}$$

In the equation, *x* represents the number of cell-cell communication based on active expressed neuropeptide-receptor pairs. *y* depicts the total cells in the dataset. Therefore, *y*^2^ is all of the potential cell-cell communication in the corresponding dataset. When the stable expression ratio is over 50%, we defined this neuropeptide-receptors pair as a stable cell communication in this dataset. Based on this criterion, we identified stable neuropeptide-receptor interaction pairs in all 70 datasets.

### 2.4. Mutational and Survival Analysis for the 39 Stably Expressed Neuropeptides and Receptors

For the 39 stably expressed neuropeptides and their receptors in multiple scRNASeq datasets, we conducted a further systematic mutational and survival analysis using a large amount of public cancer genomics data. By using cBioportal [13], we calculated the mutation patterns of these 39 genes in the 32 TCGA cancer types. At the same time, we also used these collated clinical data to conduct statistical analysis on various clinical information of the 39 gene mutations in the case samples. Suppose the case sample has survival time information. In that case, it is also used for integrated survival analysis to find evidence directly related to the prognosis and survival of various cancers. To systematically interpret the functional classification involved in these 39 genes, we used the most popular tools ToppFunc [14] and Metascape [15].

Regarding the enrichment of oncogenes and tumor suppressor genes, we downloaded all known oncogenes and tumor suppressor genes from ONGene [16] and TSGene [17]. Then the phyper function in R language was used to perform hypergeometric statistical analysis and *p*-value calculation. In this article, the neuropeptide receptor network was displayed based on Cytoscape [18].

### 2.5. Cell State and t-Distributed Stochastic Neighbour Embedding Analysis

To explore the cell state related to neuropeptides and receptors, we utilized an R package GSVA [19]. To quantify the cell state related to the neuropeptides, we used 39 stably expressed neuropeptides and receptors as signatures. To explore if those states correlated with the other cell states, we focused on a dataset with the majority of cell-cell communication based on actively expressed neuropeptides and receptors. The t-Distributed Stochastic Neighbor Embedding (t-SNE) plot was implemented by using the R package Seurat [20]. The correlations among the cell states were calculated by cor.test function in the R. The correlated cell states were plotted as vectors in the t-SNE chart with direction and quantity. Specifically, the vectors pointed toward the directions where the cell states changed. The lengths of those vectors were correlated with all signatures used for the cell state calculation.

## 3. Results

### 3.1. The Workflow of Constructing Neuropeptide and Receptor-Based Cell Communication Network

**Figure 1:**
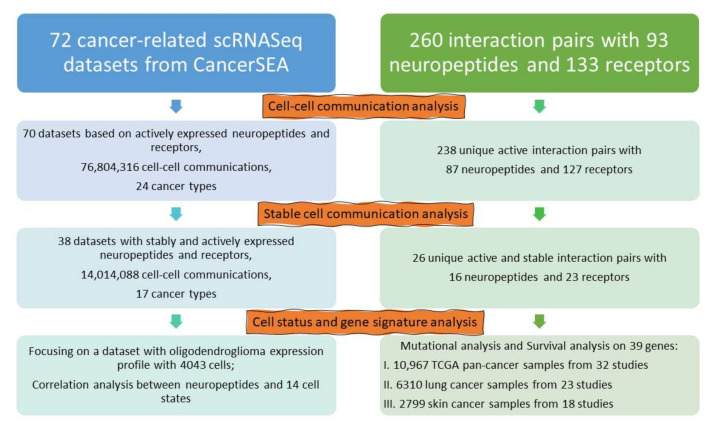
A diagram depicting the construction of a cell-to-cell communication network based on neuropeptide-receptor interactions. The results (orange) and step-by-step analyses (**left**) are organized depending on the datasets (**left**) and the number of neuropeptides (**right**). We identified actively expressed neuropeptides and receptors by cell-cell communication analysis. The stable cell communication analysis narrows down the neuropeptide and receptor connection based on the percentage of a certain neuropeptide-receptor among all possible cell-cell communications in a dataset. To cross-validate our prioritized genes, we performed an additional cell status and signature analysis using public cancer genomic datasets.

Unprecedented chances to investigate the functional heterogeneity of cancer cells are now available thanks to a significant amount of published scRNAseq transcriptome data. Our analysis pipeline is based on two types of information to detect neuropeptides and receptors-based cell communications at a single-cell level (Figure 1). One is the well-known links between neuropeptides and their receptors from our previously published work [9]. The other is single-cell transcriptome data from various malignancies. To take advantage of high-quality scRNAseq data, we downloaded the CancerSEA database, which provided uniform quantification gene expression at the single-cell level. In total, we obtained 72 data sets encompassing 25 distinct cancer types from CancerSEA.

In our published work [9], we collected a total of 260 human neuropeptide receptor interaction pairs, including 93 neuropeptides and 133 receptor genes. Dropouts, when the data only catch a small fraction of the transcriptome of each cell, are one of the challenges in scRNAseq data analysis. Almost all quantitative expressions specific for scRNAseq have used gene selection, dimension reduction, or imputation to address the dropouts. To get around this obstacle, we only looked at neuropeptides and receptors that were actively expressed in this study. By examining the expression in 72 cancer scRNASeq datasets, we discovered 87 neuropeptides and 127 receptors were actively expressed, forming 238 novel interactional pairs.

Even though there are only 214 distinct actively expressed neuropeptide and receptor genes, they have built up massive cell-to-cell communication networks. For instance, we found 76,804,316 cell-to-cell interactions in 70 datasets spanning 24 cancer types. It was necessary to eliminate two datasets since the actively expressed neuropeptides and receptors were not detected in the two datasets. The NXPH1-NRXN1 pair is the most common cell communication pair among them. In a dataset featuring oligodendroglioma (GSE70630) [21], for example, this pair has linked a total of 9,928,473 cell-to-cell communications. There are 4043 single cells in this dataset, which might result in 16,345,849 (4043 times 4043) cell-to-cell exchanges theoretically. As a result, we may assume that *NXPH1*-*NRXN1* is involved in almost 61% (9,928,473 divided by 16,345,849) of all conceivable cell-to-cell communications in the dataset.

Identifying stable and reproducible expression across a range of abundances may be helpful to score gene signatures and normalize transcriptomic data across cells [22]. We defined stable cell communication as a ratio of neuropeptide-based cell communication to all conceivable cell-to-cell contacts that is greater than 50%. In a word, the stably expressed neuropeptide-receptor pair must be steady in half or more of the potential cell-to-cell communications.

By applying this criterion to all the 70 datasets, we only identified 26 unique actively and stably expressed neuropeptide-receptor pairs. In fact, stable cell communication analysis also narrowed down the number of scRNASeq datasets from 70 to 38. In addition, we defined 39 genes (16 neuropeptides and their 23 receptors) as the stably expressing neuropeptide network involved in 14,014,088 cell-to-cell communications across 17 cancer types. Due to the importance of these 39 genes, our subsequent analysis conducted a more in-depth signature and survival analysis.

### 3.2. The Consistent Neuropeptide Initiated Cell-to-Cell Communications across Multiple Cancers

**Figure 2:**
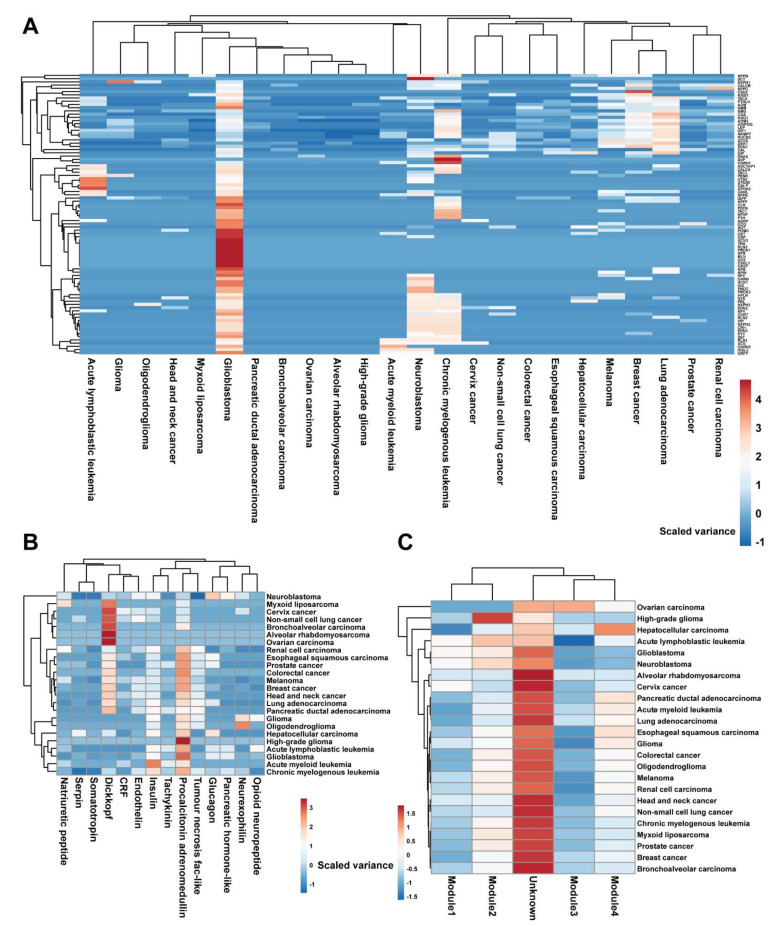
The overview of neuropeptide-receptor-based cell communication. (**A**) The heatmap depicts the number of cell-to-cell communications initiated by 89 neuropeptides (rows) in 24 cancer types (columns). (**B**) The heatmap depicts the total cell-to-cell communications initiated by 14 neuropeptide families (columns) among 24 cancer types (rows). (**C**) Across 24 cancer types, the heatmap shows the cell connections initiated by four neuropeptide-receptor functional modules (columns) (rows).

To explore the role of neuropeptides at the single-cell level, we compared the cell communications initiated by all neuropeptides across 24 cancer types (Figure 2A). All neuropeptides implicated, in general, perform varied functions in various cancer types. Interestingly, brain and central nervous system (CNS) cancers exhibit relatively more active neuropeptides and receptors. The most notable feature is that almost all neuropeptides are expressed in GBM and have receptors for signal transduction. There are 3 GBM datasets, which have 279 neuropeptide-receptor interactions initiated by 84 neuropeptides. In addition, another 275 neuropeptide-receptor pairs are detected in leukemia datasets. There are also sporadic interactions specific to breast cancer and lung cancer. In summary, this overview presentation confirmed that neuropeptides are important in the brain and CNS cancer development.

By classifying these neuropeptides according to the protein family, we have statistics about the unique active neuropeptides in 24 cancer types (Figure 2B). In practice, we require a neuropeptide family with a minimum of three neuropeptide genes. As shown in Figure 2B, 15 neuropeptide families are summarized. The most prominent family is the insulin family with seven neuropeptides. We then eliminated the redundant neuropeptide in different cells and focused on the unique neuropeptides in each dataset. In total, we found 238 non-redundant neuropeptide receptor interactions include 84 neuropeptides. More interestingly, we found all neuropeptides are activated in the glioblastoma cohort. Procalcitonin and adrenomedullin family is one of the most widely expressed neuropeptide families. Not only are procalcitonin/adrenomedullin neuropeptides found in GBM, but they are also found in 16 other cancers. Similarly, the neuropeptides belonging to the Dickkoff family are also widely active expression in 16 cancer types.

Based on our previously identified functional module, we further compared the expression pattern of different modules among various cancer types. We discovered that diverse forms of cancer are influenced by a vast number of disconnected (unknown module) neuropeptides and receptors. We might also be able to uncover some cancer type-specific modules. Glioma, for example, has a high concentration of module 2. Module 2 has 39 genes, including 15 neuropeptides and 24 receptors, with the *CORT* neuropeptide being the most linked. Module4 is also highly expressed and plays a critical function in liver cancer. Module 4 has 17 genes, including 6 neuropeptides such as *KNG1*. Consequently, a substantial number of neuropeptides and their receptors have a strong cancer-type specificity in their expression. In sum, there are a lot of neuropeptide receptors that cannot be categorized by neuropeptide family or functional module. This could also mean that those unclassified neuropeptides may regulate cellular processes through orphan receptors.

### 3.3. The 39 Neuropeptides and Receptors Connected over Half Cell in Each Dataset

**Figure 3:**
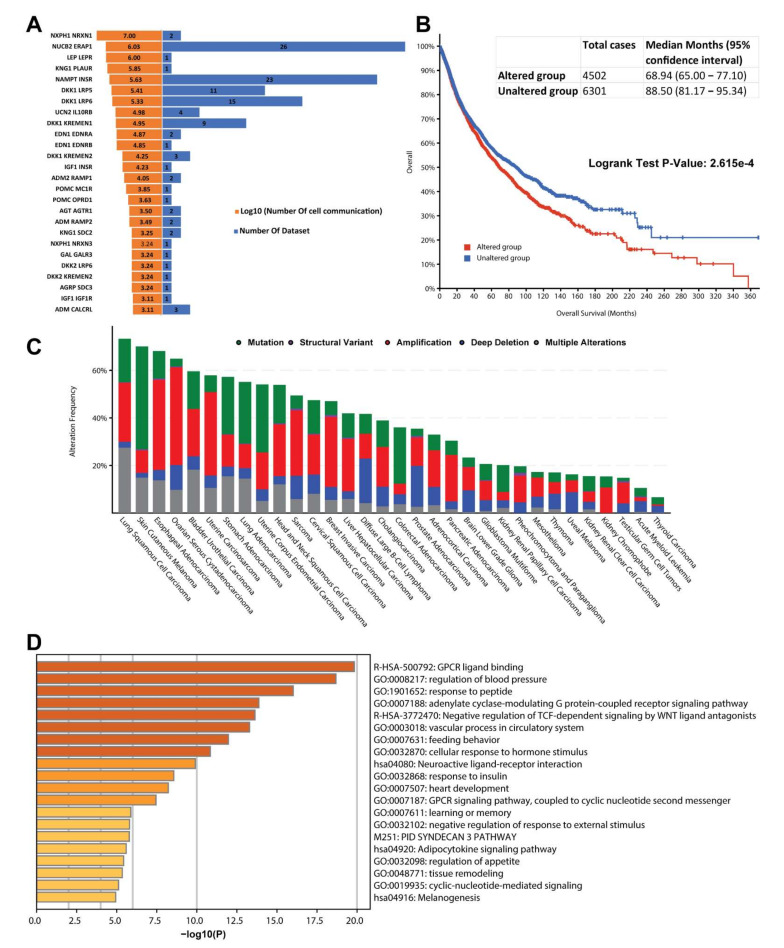
The functional feature of stably expressed neuropeptide-receptor genes. (**A**) The butterfly chart depicts the number of cell communications and datasets associated with the 26 stably expressed neuropeptide-receptor pairs. (**B**) The Kaplan–Meier curves show the overall survival months for patients with specific mutations (red) and no mutations (blue) in each of the 39 genes. (**C**) The vertical bar chart depicts the frequency of mutations in 32 TCGA cancer types. (**D**) The functional cluster representatives for the 39 genes are summarized in the bar plot. The x-axis indicates the log of corrected *p*-value calculated by the hypergeometric test. The darker color represents more significant *p*-values.

In the first stage, we built 76,804,316 cell-to-cell interactions from 70 single-cell expression datasets from cancer patients. We are now ready to execute the stable expression analysis in order to focus on reproducible and stable cell communications. Each neuropeptide-receptor interaction must be observed in more than half of all conceivable cell communications, according to the criteria. The square of cells in a dataset was used to represent all conceivable cell communications. Then, we determined how extensive each neuropeptide-receptor pair is by comparing the cell communication initiated by each neuropeptide-receptor pair. As a result, we were able to decrease the original 70 datasets to just 38. In detail, 16 neuropeptides and 23 receptors were primarily responsible for those cell communications (Figure 3). It is worth noting that if the stable expression ratio is more than 0.5, it signifies that these neuropeptide-receptors were expressed actively and consistently in the majority of the cells in the dataset.

As shown in Figure 3A, NXPH1-NRXN1 was the leading interaction responsible for signal transduction between cells. This neuropeptide-receptor pair involved a total of 9,930,195 cell-to-cell connections in 2 data sets. One of them is the aforementioned oligodendroglioma dataset (GSE70630) [21]. The other is in the cervix cancer cell line data set (GSE101519) [23]. NXPH1 and its two downstream receptors, NRXN1 and NRXN3, have established 1722 cell-to-cell communications, accounting for 97.6% of all possible cell-cell links in cervix cancer cell lines. Interestingly, we could not find NXPH1-NRXN3 in the oligodendroglioma dataset. In sum, our results may indicate a certain degree of cell type-specific receptor expression patterns.

To further assess the potential functions for these 39 neuropeptides and receptors, we utilized the TCGA pan-cancer genomics data to examine if they are related to patient prognostic features. Of the 10,803 patients from 32 cancer cohorts, 4502 cases have specific genetic mutations among these 39 genes. The median survival time is about 68.94 months compared to 88.50 months from the 6301 cancers without mutations in the 39 genes. The log-rank test *p*-value is about 2.615 × 10^−4^, indicating that 39 genes are significantly associated with patient survival across multiple cancer types. As shown in Figure 3C, four cancer cohorts have over 60% of patients with mutated genes, including lung squamous cell cancer (LUSC), melanoma, esophageal adenocarcinoma, and high-grade ovarian cancer. Interestingly, the lung adenocarcinoma cohort has a relatively lower mutational rate. Unexpectedly, two brain tumors (glioblastoma and low-grade glioma) were just about 30% mutation frequency among these 39 genes.

The additional functional enrichment analysis was performed to examine the key molecular mechanisms underlying these 39 genes. As shown in Figure 3D, these genes are enriched in GPCR-ligand binding and response to the peptide as expected. Strikingly, we also found 19 genes associated with blood pressure regulation (Adjusted *p*-value = 1 × 10^−18.68^). As a common side effect of cancer treatment, high blood pressure is particularly important for chemotherapy and targeted therapy. And these genes are also crucial for heart development, response to insulin, learning, and memory.

By focusing on the mutational frequency of each gene (Figure S1, Table S4), we could find 17 genes mutated in over 200 cancer patients, which is unlikely to have occurred by chance. For example, KNG1 was mutated in 690 patients across 25 cancer types, accounting for 6% of the 10,803 patients. Another 4 genes mutated in over 400 samples are *NRXN1* (595 samples), *LRP5* (529 samples), *NRXN3* (452 samples), and *SDC2* (437 samples). We could detect mutations on *NRXN1* and *NRXN3* in 950 cancer samples across 32 cancer types. Generally speaking, this result may indicate the importance of the neurexins family in various cancer development. Our cell communication evidence at the single-cell level may provide the potential mechanisms underlying the neurexins.

### 3.4. Survival and Mutational Analysis for the 39 Top-Ranked Genes

**Figure 4:**
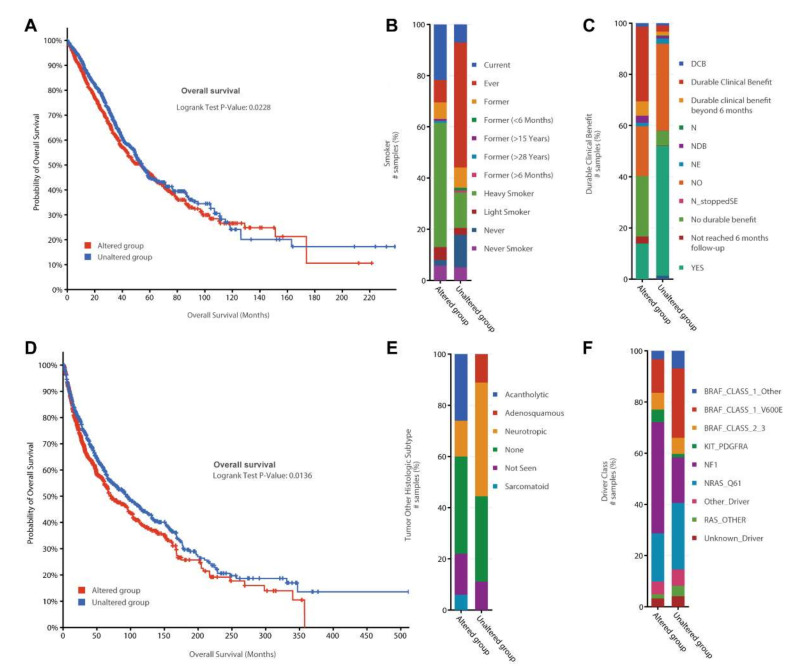
The survival and clinical feature analyses for the 39 stably expressed neuropeptides and receptors in 23 lung cancer studies (**A**–**C**) and 18 skin cancer studies (**D**–**F**). (**A**) The Kaplan–Meier curves show the overall survival months for 6310 lung cancer patients with specific mutations (red) and no mutations (blue) in 39 genes. (**B**) The stacked bar chart shows whether or not each of the 6310 samples smoked. (**C**) A stacked bar chart depicts long-term clinical benefits distribution in 6310 lung cancer samples. (**D**) The Kaplan–Meier curves show the overall survival months for 1063 skin cancer samples with specific mutations (red) and no mutations (blue) in the 39 genes. (**E**) The histological subtypes for all 1063 samples are summarized in the stacked bar chart. (**F**) A stacked bar chart shows how the driver mutations in 1063 skin cancer samples were distributed.

Since we found the lung cancer and melanoma have the highest mutational rate in 39 genes, our second set of analyses were focused on these two cancer types (Figure 4). By expanding the cancer types to multiple independent datasets, we examine the consistent features for our 39 genes. In detail, we integrated 6310 samples in 23 lung cancer studies for extensive genomic and clinical feature investigation (Figure 4A–C). As expected, the combined data have significant survival analysis *p*-values (Figure 4A), which confirmed our result from the TCGA pan-cancer analysis. More interestingly, we found many significantly different clinical features among the cohorts with and without any mutations in our prioritized 39 genes. As an example, in Figure 4B the lung cancer patients showed distinct smoking history between the patients with and without mutations in our 39 genes. The patient group, with relatively short survival time and mutations in the 39 genes, have a high proportion of current smoker and former smoker (less than six months). More interestingly, durable clinical benefit (DCB, immunotherapy efficacy time to progression or next treatment of more than six months) was observed in about 30% of patients with mutations on the 39 genes (Figure 4C). This might suggest those 39 genes are critical to improve immunotherapy for lung cancer patients. In terms of somatic mutations, we found four genes highly mutated in over 200 samples: *NRXN1* (470 samples, ~13%), *KNG1* (441 samples ~12%), *AGTR1* (264 samples, ~7%), and *NRXN3* (259 samples, ~7%).

Similarly, another 2799 skin cancer samples were combined from 18 studies using cBioPortal (Figure 4D–F). Of the combined population, 866 patients have some genetic mutations on the 39 genes, which only have a median survival month of 71.80 compared to 94.61 months for 1063 patients without any mutations (Figure 4D). By further investigating the histologic subtypes, we found that the altered group has more acantholytic cases while the unaltered group enriched neurotropic cases (Figure 4E). The driver mutation distribution also revealed the genetic difference between the two groups. There are more neurofibromin 1 (NF1) mutations in the 866 altered patients. On the contrary, the 1063 patients from the unaltered group are more likely to mutate in BRAF (V600E) and NRAS (Q61). By examining the gene mutational frequency, three genes are mutated in over 200 samples: NRXN1 (295 samples, ~16%), NRXN3 (223 samples, ~12%), and LEPR (205 samples, ~11%). Taken together, the distinct histological feature and the molecular driver may imply the completely different mechanisms for neuropeptide-involved skin cancers.

### 3.5. Neuroppeptides and Receptors-Based Cell Status

We further assessed each cell sample’s neuropeptide receptor status characteristics in the 38 data sets involved using the 39 genes. In addition, the neuropeptide-receptor status was correlated to the other cell states systematically. Since *NXPH1*-*NRXN1* is the leading interaction associated with most cell communications, we used the oligodendroglioma dataset as an example to demonstrate the neuropeptide-receptor cell status (GSE70630) [21].

**Figure 5:**
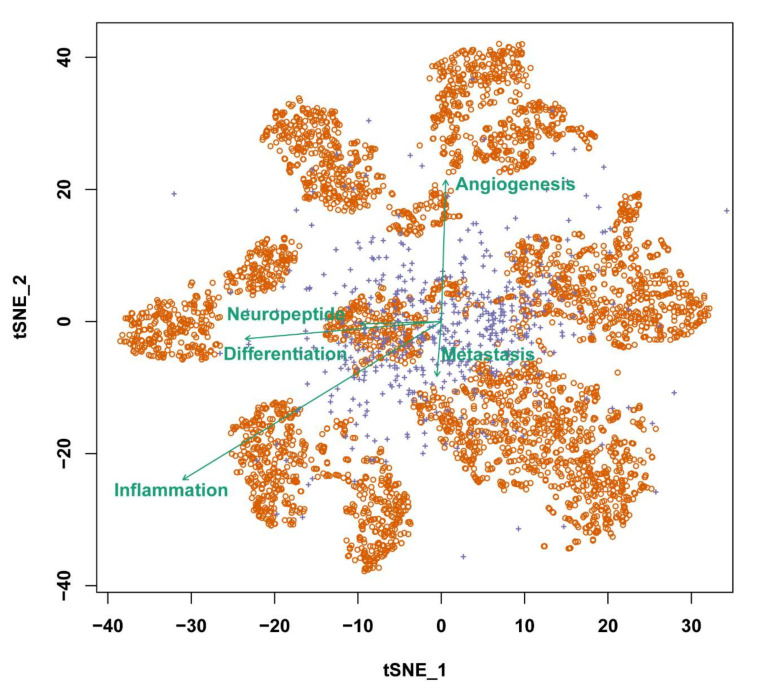
The t-SNE biplot demonstrates the connection between intratumor heterogeneity and cell status in 4825 oligodendroglioma cells. The orange circles represent cells, whereas the purple “+” represents the genes with the highest percentage of variants. Green vectors represented the five cell states.

As shown in Figure 5, in oligodendroglioma cells, the neuropeptide had a strong relationship with differentiation status. In general, cells can be split into eight groups. The metastatic and angiogenesis vectors were virtually perpendicular to the neuropeptide status vector, but had the same direction. Taken together, this shows that these neuropeptides and receptors may play a role in cell differentiation rather than angiogenesis and metastasis in the formation of oligodendroglioma. Since neuropeptides and receptors surround the cell membrane, they may affect the tumor microenvironment. Finally, these 13 neuropeptides and 26 receptors may be valuable indicators for evaluating disease progression due to tumor heterogeneity.

## 4. Discussion

To summarize, we have for the first time investigated the interplay of neuropeptides and their receptors in diverse malignancies at the single-cell level. Our findings revealed not only the first comprehensive neuropeptide single-cell interaction map but also 39 valuable biomarker genes that can be exploited to detect cancer prevalence and progression at the single-cell level. We may examine how active neuropeptide-initiated communications are by identifying a set of stably expressed neuropeptide-receptor couples whose expression levels and communication broadness should not vary considerably under different experimental settings. We discovered that 39 stably expressed neuropeptides and their receptor genes are directly associated to prognostic and survival indicators of diverse malignancies after integrating a massive amount of cancer genomics data. These methods may provide a clear direction for the ongoing development of neuropeptide-based pharmacological treatments since neuropeptides and their receptors can deliver novel hormone-based treatments with low adverse effects.

To reconstruct the communication network between cells, we only looked at neuropeptides with identified receptors. As a result, neuropeptides with unknown receptors may go unnoticed. In addition, the ligand-receptor data we used was published in 2015 [24]. Therefore, we may also miss some newly identified neuropeptide-receptor information in the last six years. From the primary results of this study, we can find that a single neuropeptide and its receptor can form millions of cell connections. As a result, each newly discovered neuropeptide receptor interaction will alter the intercellular communication network at the single-cell level. Therefore, we anticipate that a more comprehensive neuropeptide-receptor interaction will significantly increase the cellular communication network. Another interesting finding is that neuropeptides and their receptors have extreme specificity on cancer type. This also means that we also overlooked some cancer-specific neuropeptide-initiated communication if the single-cell transcriptomes are not available for certain cancers. We can only look forward to a more comprehensive and standardized human cancer single-cell system map in response to this defect.

A reference list of stably expressed genes will be valuable for expression normalization in transcriptome analysis [25]. This study defined stably expressed pairings that included both active neuropeptide expression and receptors. Similarly, we believe that these persistent neuropeptide-receptor combinations might be utilized as a benchmark for determining how fast and extensive cell communication is. As a result, cell communication pair-based normalization will effectively compensate for disparities in the technical platform or sample size.

Since 1961, evidence has accumulated indicating neurotransmitters and peptides such as vasopressin, neurotensin, thyrotropin-releasing hormone, somatostatin, substance P, enkephalins, opioid peptide, and endorphin may play a role in blood pressure management [26]. Recently, a new interdisciplinary called cardio-oncology was established with the goal of ensuring proper heart health monitoring throughout oncogenic treatment [27]. We predict more and more ties between neuropeptides and cardio-oncology research as a result of our findings, which may emphasize the critical role of neuropeptides in cardiovascular management [28].

One of the most important features of our analysis is the stable and active expression of neurexins (*NRXN1* and *NRXN3*). The *NRXN1* with epidermal growth factor-like (EGF-like) domain has been shown to interact with neurexophilins [29]. In general, their proteins are cell adhesion molecules located at presynaptic regions and associated with neurodevelopmental disorders. For instance, *NRXN3* has been associated with alcohol or drug addiction [30]. It is assumed that the mutated neurexins may disturb the synaptic stabilization and function [31]. Our analysis may highlight their important role in cancer development by initiating cancer cell communications. In addition, they are highly mutated in various cancers such as lung cancers and skin cancers. In summary, they might be good candidate therapeutic targets in multiple cancers.

Despite the fact that our analysis revealed a link between neuropeptides and smoking/long-term clinical benefit, no attempt is made to provide a complete examination of the significance of these clinical factors in cancer development. Our findings are unique in that they are the first to show a significant difference in smoking/durable clinical benefit status between lung cancer patients with and without mutations in specific neuropeptides and receptors. In thousands of skin cancer cases, we highlighted distinctions in histological subgroups (acantholytic vs. neurotropic) and driver mutational patterns.

## Data Availability

The raw data used were from public Gene Expression Omnibus databases: GSE65525 and GSE70630.

## Supplementary Materials

The following are available online at https://www.mdpi.com/article/10.3390/biomedicines10010014/s1. Figure S1: The sample-based oncoprint of mutational patterns for 39 genes in 6031 lung cancer samples combined from 26 studies; Table S1: 260 neuropeptide and receptor interactions used to construct cell communications in cancer cells; Table S2: Four neuropeptide and receptor interaction modules; Table S3: The 70 single-cell transcriptome datasets used in this study; Table S4: The mutational frequency of the stably expressed 39 neuropeptides and receptors in TCGA pan-cancer cohort.
