# Supplementary material for: Decode the Stable Cell Communications Based on Neuropeptide-Receptors Network in 36746 Tumor Cells
Source: Biomedicines. 2021 Dec 22;10(1):14. doi: 10.3390/biomedicines10010014 (PMC8773225; doi:10.3390/biomedicines10010014)
Supplement: Supplementary file 1 [file FigureS1_39TCGApan_oncoprint.pdf › Supp/FigureS1_39TCGApan_oncoprint.pdf]

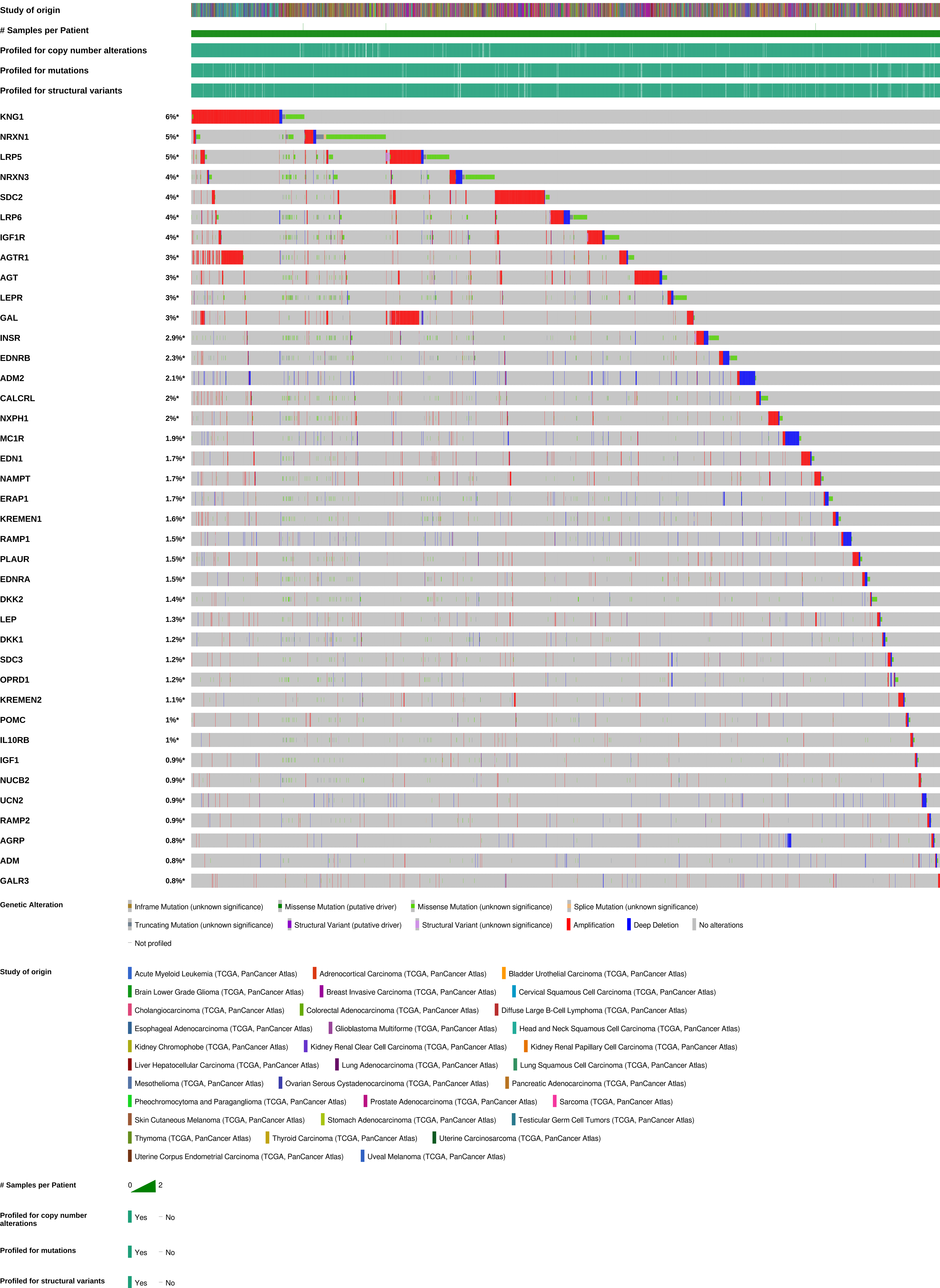
Study of origin
Samples per Patient
Profiled for copy number alterations
Profiled for mutations
Profiled for structural variants
KNG1 6%*
NRXN1 5%*
LRP5 5%*
NRXN3 4%*
SDC2 4%*
LRP6 4%*
IGF1R 4%*
AGTR1 3%*
AGT 3%*
LEPR 3%*
GAL 3%*
INSR 2.9%*
EDNRB 2.3%*
ADM2 2.1%*
CALCRL 2%*
NXPH1 2%*
MC1R 1.9%*
EDN1 1.7%*
NAMPT 1.7%*
ERAP1 1.7%*
KREMEN1 1.6%*
RAMP1 1.5%*
PLAUR 1.5%*
EDNRA 1.5%*
DKK2 1.4%*
LEP 1.3%*
DKK1 1.2%*
SDC3 1.2%*
OPRD1 1.2%*
KREMEN2 1.1%*
POMC 1%*
IL10RB 1%*
IGF1 0.9%*
NUCB2 0.9%*
UCN2 0.9%*
RAMP2 0.9%*
AGRP 0.8%*
ADM 0.8%*
GALR3 0.8%*
Genetic Alteration
Inframe Mutation (unknown significance)
Missense Mutation (putative driver)
Missense Mutation (unknown significance)
Splice Mutation (unknown significance)
Truncating Mutation (unknown significance)
Structural Variant (putative driver)
Structural Variant (unknown significance)
Amplification
Deep Deletion
No alterations
Not profiled
Study of origin
Acute Myeloid Leukemia (TCGA, PanCancer Atlas)
Adrenocortical Carcinoma (TCGA, PanCancer Atlas)
Bladder Urothelial Carcinoma (TCGA, PanCancer Atlas)
Brain Lower Grade Glioma (TCGA, PanCancer Atlas)
Breast Invasive Carcinoma (TCGA, PanCancer Atlas)
Cervical Squamous Cell Carcinoma (TCGA, PanCancer Atlas)
Cholangiocarcinoma (TCGA, PanCancer Atlas)
Colorectal Adenocarcinoma (TCGA, PanCancer Atlas)
Diffuse Large B-Cell Lymphoma (TCGA, PanCancer Atlas)
Esophageal Adenocarcinoma (TCGA, PanCancer Atlas)
Glioblastoma Multiforme (TCGA, PanCancer Atlas)
Head and Neck Squamous Cell Carcinoma (TCGA, PanCancer Atlas)
Kidney Chromophobe (TCGA, PanCancer Atlas)
Kidney Renal Clear Cell Carcinoma (TCGA, PanCancer Atlas)
Kidney Renal Papillary Cell Carcinoma (TCGA, PanCancer Atlas)
Liver Hepatocellular Carcinoma (TCGA, PanCancer Atlas)
Lung Adenocarcinoma (TCGA, PanCancer Atlas)
Lung Squamous Cell Carcinoma (TCGA, PanCancer Atlas)
Mesothelioma (TCGA, PanCancer Atlas)
Ovarian Serous Cystadenocarcinoma (TCGA, PanCancer Atlas)
Pancreatic Adenocarcinoma (TCGA, PanCancer Atlas)
Pheochromocytoma and Paraganglioma (TCGA, PanCancer Atlas)
Prostate Adenocarcinoma (TCGA, PanCancer Atlas)
Sarcoma (TCGA, PanCancer Atlas)
Skin Cutaneous Melanoma (TCGA, PanCancer Atlas)
Stomach Adenocarcinoma (TCGA, PanCancer Atlas)
Testicular Germ Cell Tumors (TCGA, PanCancer Atlas)
Thymoma (TCGA, PanCancer Atlas)
Thyroid Carcinoma (TCGA, PanCancer Atlas)
Uterine Carcinosarcoma (TCGA, PanCancer Atlas)
Uterine Corpus Endometrial Carcinoma (TCGA, PanCancer Atlas)
Uveal Melanoma (TCGA, PanCancer Atlas)
Samples per Patient
0
2
Profiled for copy number alterations
Yes
No
Profiled for mutations
Yes
No
Profiled for structural variants
Yes
No
